# A Cell Permeable Peptide Inhibitor of NFAT Inhibits Macrophage Cytokine Expression and Ameliorates Experimental Colitis

**DOI:** 10.1371/journal.pone.0034172

**Published:** 2012-03-27

**Authors:** Houda Z. Elloumi, Nitsan Maharshak, Kavitha N. Rao, Taku Kobayashi, Hyungjin S. Ryu, Marcus Mühlbauer, Fengling Li, Christian Jobin, Scott E. Plevy

**Affiliations:** 1 Center for Gastrointestinal Biology and Diseases, Departments of Medicine and Department of Microbiology and Immunology, University of North Carolina School of Medicine, Chapel Hill, North Carolina, United States of America; 2 Department of Immunology, University of Pittsburgh School of Medicine, Pittsburgh, Pennsylvania, United States of America; University of North Dakota, United States of America

## Abstract

Nuclear factor of activated T cells (NFAT) plays a critical role in the development and function of immune and non-immune cells. Although NFAT is a central transcriptional regulator of T cell cytokines, its role in macrophage specific gene expression is less defined. Previous work from our group demonstrated that NFAT regulates *Il12b* gene expression in macrophages. Here, we further investigate NFAT function in murine macrophages and determined the effects of a cell permeable NFAT inhibitor peptide 11R-VIVIT on experimental colitis in mice. Treatment of bone marrow derived macrophages (BMDMs) with tacrolimus or 11R-VIVIT significantly inhibited LPS and LPS plus IFN-γ induced IL-12 p40 mRNA and protein expression. IL-12 p70 and IL-23 secretion were also decreased. NFAT nuclear translocation and binding to the IL-12 p40 promoter was reduced by NFAT inhibition. Experiments in BMDMs from IL-10 deficient (*Il10* ^−/−^) mice demonstrate that inhibition of IL-12 expression by 11R-VIVIT was independent of IL-10 expression. To test its therapeutic potential, 11R-VIVIT was administered systemically to *Il10* ^−/−^ mice with piroxicam-induced colitis. 11R-VIVIT treated mice demonstrated significant improvement in colitis compared to mice treated with an inactive peptide. Moreover, decreased spontaneous secretion of IL-12 p40 and TNF in supernatants from colon explant cultures was demonstrated. In summary, NFAT, widely recognized for its role in T cell biology, also regulates important innate inflammatory pathways in macrophages. Selective blocking of NFAT via a cell permeable inhibitory peptide is a promising therapeutic strategy for the treatment of inflammatory bowel diseases.

## Introduction

Macrophages perform numerous critical functions during immune responses such as phagocytosis, microbial killing, antigen presentation to T cells, and production of inflammatory mediators. Microbial stimulation of macrophages through toll-like receptors (TLRs) leads to a cascade of signaling events culminating in the production of various inflammatory cytokines including IL-12, IL-23 and TNF; and antimicrobial mediators such as free oxygen radicals, nitric oxide (NO) and proteases. Expression of these mediators needs to be tightly regulated, as failure could contribute to the pathogenesis of chronic inflammation in diseases including rheumatoid arthritis, multiple sclerosis and inflammatory bowel diseases (IBD) [1].

The IL-12 family of cytokines has been implicated in the pathogenesis of numerous chronic inflammatory disorders [2], [3], [4], [5]. IL-12 and IL-23 are heterodimeric cytokines composed of a common p40 subunit in addition to p35 and p19 subunits, respectively. IL-12 is involved in the development of a T-helper-1 (Th1) response [6], [7]. IL-23 is involved in the maintenance of Th17 cells [8]. IL-12 p40 is expressed specifically in macrophages and dendritic cells [9] and is highly induced by microbial constituents such as LPS, CpG rich bacterial DNA, lipoproteins and T-cell dependent cognate interactions [7]. Detailed molecular characterization of IL-12 p40 (*Il12b*) promoter activation by microbes and cytokines has described complex regulation through numerous protein-DNA and protein-protein interactions [10], [11], [12]. Our group described a novel composite element in the *Il12b* promoter that interacts with members of the nuclear factor of activated T cells (NFAT) and the interferon regulatory factor (IRF) families of transcription factors [10]. This element is involved in the synergistic induction of *Il12b* promoter activity by bacterial products and interferon-gamma (IFN-γ).

The NFAT family of transcription factors plays a key role in cytokine gene expression in T cells. Five NFAT family members have been identified, four of which are calcium-regulated and require the calcium/calmodulin-dependent phosphatase, calcineurin, for their nuclear localization. The role of NFAT in immune responses has been best characterized in T cells. Upon T cell activation by antigen, intracellular calcium levels increase and calcineurin is consequently activated. This serine/threonine phosphatase activates cytosolic NFAT through its dephosphorylation which results in activated NFAT translocation to the nucleus and in inflammatory cytokines production. NFAT has also been described to mediate gene expression in other cells of the immune system and other organ systems [13]. However, less is known about NFAT function in other cells of the immune system, specifically macrophages, although its role in related cell types such as dendritic cells and osteoclasts is under intensive investigation over the past few years [14], [15], [16], [17]. Moreover, calcineurin inhibitors have been reported to inhibit IL-12 p40 expression in human monocytic cells [18].

Calcineurin inhibitors, such as tacrolimus and cyclosporine (CsA), bind to specific binding proteins (FK506-binding proteins and cyclophilins, respectively) and these complexes attach to calcineurin and prevent its dephosphorylation-induced NFAT activation. These agents revolutionized transplantation medicine through prevention of graft rejection and consequent dramatic improvement in patient survival [19], [20], [21]. Their use has been extended to chronic inflammatory diseases such as the inflammatory bowel diseases and psoriasis [22].

In contrast to the calcineurin inhibitors, a short peptide, VIVIT, inhibits NFAT activation through interaction with the calcineurin binding site for NFAT and thus prevents nuclear translocation without affecting calcineurin phosphatase activity [23]. Protein transduction domains (PTD), such as polyarginines and the HIV Tat peptide, have been utilized to facilitate the delivery of various cargo molecules into a variety of cells. PTD-mediated VIVIT delivery has demonstrated blockade of NFAT and therapeutic efficacy in various murine inflammatory disease models [24], [25]. In this study, we used the cell permeable peptide 11R-VIVIT to characterize the role of NFAT in macrophage-specific gene expression. VIVIT attenuated LPS or LPS plus IFN-γ induced IL-12 p40 mRNA and protein expression in bone marrow derived macrophages (BMDMs) and reduced DNA binding of NFAT to a composite NFAT–interferon stimulated response element (ISRE) on the *Il12b* promoter. In addition, VIVIT inhibited the production of NO and the secretion of IL-12 p70, IL-23, and TNF suggesting a global role for NFAT in inflammatory gene expression in macrophages. Furthermore, as an in vivo correlate, 11R-VIVIT ameliorated active colitis in piroxicam-treated IL-10 deficient (*Il10* ^−/−^) mice. Histologic improvement correlated with reduced spontaneous secretion of colonic inflammatory cytokines including IL-12 p40.

## Materials and Methods

### Mice

Seven to ten weeks old C57/BL6 and *Il10* ^−/−^ mice on a C57/BL6 background were purchased from the Jackson Laboratories. All animals were housed in specific pathogen free conditions (SPF) in accordance with guidelines from the American Association for Laboratory Animal Care and Research. *Il10^−/−^;*NF-κB^EGFP^ reporter mice (129/SvEv/C57BL6 background) were generated by Christian Jobin as previously described [26]. The Institutional Animal Care and Use Committee of the University of North Carolina approved all animal protocols (*Permit Number: 06-239*). To study the effects of the cell permeable inhibitor peptide 11R-VIVIT, the non-steroidal anti-inflammatory piroxicam (200 ppm) was added to the mice diet for 14 days as a well validated approach to accelerate the development of colitis in *Il10* ^−/−^ mice [27]. After the establishment of colitis and withdrawal of piroxicam from the diet, sex and age- matched *Il10* ^−/−^ mice were randomized to receive 11R-VIVIT or the control peptide 11R-VEET were delivered by intra-peritoneal injection every other day for 2 weeks. At the end of treatment period, the mice were euthanized and the colons were immediately removed.

### Reagents

Lipopolysaccharide (LPS) from *Salmonella enteritidis* was purchased from Sigma (St. Louis, MO). Tacrolimus was purchased from Alexis Biochemicals (San Diego, CA). Recombinant murine IFN-γ was purchased from R&D Systems. M-CSF was obtained from Peprotech Inc (Rocky Hill, NJ). Antibodies to NFATc1 (K18) and IRF8 (H-70) were purchased from Santa Cruz Biotechnology (Santa Cruz, CA).

### Peptide synthesis

11R-VIVIT (RRRRRRRRRRRGGGMAGPHPVIVITGPHEE) and 11R-VEET (RRRRRRRRRRRGGGMAGPPHIVEETGPHVI) peptides were synthesized and HPLC-purified (>95%) by GenScript (Piscataway, NJ) and at the peptide synthesis facilities of the University of North Carolina and the University of Pittsburgh or purchased through EMD Bioscience. 11R-VIVIT from EMD Bioscience will be referred to as VIVIT^EMD^ since this VIVIT was active at significantly lower concentrations compared to the VIVIT from GenScript.

### Bone marrow macrophage culture

Bone marrow derived macrophages (BMDMs) were prepared as described [28].

### EGFP Imaging

Epifluorescence microscopy was used to detect EGFP in bone marrow derived macrophages derived from cis-NF-κB^EGFP^ mice. EGFP expression was imaged using an Olympus IX70 (Olympus, Melville, NY) fitted with EGFP-specific filters (XF116-2; Omega Optical). Images were captured using a digital SPOTM camera (Diagnostic Instruments, McHenry, IL). Identical exposure times were used for each data point within an individual experiment. EGFP positive cells per high power field were counted and were expressed as a percentage of total number of cells.

### Electrophoretic mobility shift assays

Nuclear extracts from BMDMs were prepared using the NE-PER kit from Pierce Biotechnology, following the manufacturer's instructions. The sequence of the EMSA probe spanning the NFAT/IRF8 site of the IL-12 p40 promoter is 5′-gatcTCAGTTTCTACTTTGGGTTTCCATCAGAAAGT. EMSAs were performed as described previously [10].

### Real-time RT-PCR

Total RNA was extracted using either the TRIZOL reagent (Invitrogen, Carlsbad, CA) or the RNeasy mini kit (Qiagen, Valencia, CA). Real-time RT-PCR was performed as described previously [29] to detect *Il12b* and *Nos2* mRNA.

### Cytokine ELISA

Murine IL-12 p40, IL-12 p70, IL-10, interferon-γ and TNF immunoassay kits (R & D Systems, Minneapolis, MN) were used according to manufacturers' instructions.

### Nitrite Determination

Nitrite concentration was assayed by a standard Greiss Reaction adapted to a microplate system, as described previously [28].

### T cell purification and activation

Splenic CD4+ T cells were purified from WT mice using CD4 (L3T4) Microbeads (Miltenyi Biotec) then stimulated with plate bound anti-CD3 (5 µg/ml) and anti-CD28 (2 µg/ml) (eBioscience) and exposed to various concentrations of 11R-VIVIT^EMD^ or VEET. Cells were harvested after 4 hours for mRNA and supernatants were collected after 48 hours for ELISA.

### Colonic tissue explant culture and histology

Colonic explant cultures were performed as described previously [29]. Fixed and paraffin embedded colonic sections were stained with hematoxylin and eosin (H&E). Sections were then visualized by light microscopy and histologic scores determined by three independent experienced investigators blinded to treatment group. Colitis severity index was determined based on standard criteria for inflammation, edema, hyperplasia, and atrophy as previously described [30].

### Statistical analysis

All results are expressed as mean ± SEM/SD. Using GraphPad Prism software, Mann Whitney and unpaired student's *t* test were used to assess statistical significance where appropriate.

## Results

### NFAT regulates *Il12b* gene expression

**Figure 1:**
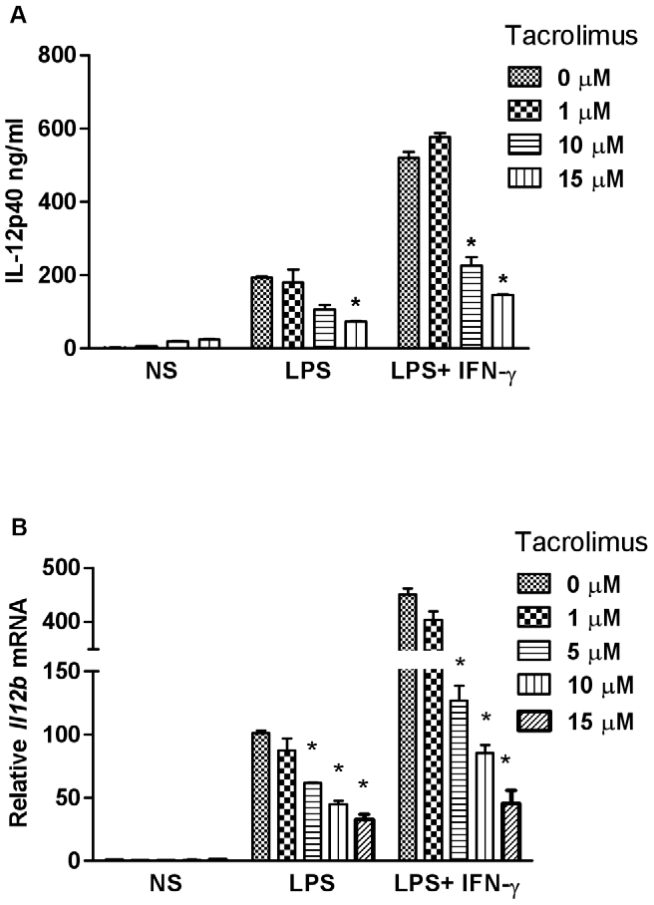
Tacrolimus inhibits IL-12 p40 protein and mRNA expression. (**A**) Murine bone marrow-derived macrophages (BMDMs) were pretreated with the indicated concentrations of tacrolimus for 1 h. Cells were then either left untreated (NS) or stimulated with LPS (100 ng/ml) or LPS and IFN-γ (10 ng/ml) for 24 h. IL-12 p40 protein secretion was assayed from supernatants by ELISA. (**B**) BMDMs were incubated with the indicated concentrations of tacrolimus for 1 h followed by 1 h treatment with IFN-γ (10 ng/ml, where indicated) prior to LPS (100 ng/ml) stimulation for 4 h or left untreated (NS). Cells were harvested and total RNA was assayed for *Il12b* mRNA levels by real-time RT-PCR. Each result represents the mean ± standard error (SD) for duplicate assays from three independent experiments. * p<0.05.

We have previously demonstrated a composite NFAT DNA binding site/interferon stimulated response element in the *Il12b* promoter [10]. To investigate the functional role of NFAT in *Il12b* expression, we utilized the well characterized and clinically relevant NFAT inhibitor tacrolimus. In bone marrow derived macrophages (BMDMs) stimulated with LPS alone or LPS plus IFN-γ, IL-12 p40 protein secretion was inhibited by tacrolimus in a dose dependent manner (Figure 1A). *Il12b* mRNA also demonstrated similar dose-dependent inhibition by tacrolimus (Figure 1B).

**Figure 2:**
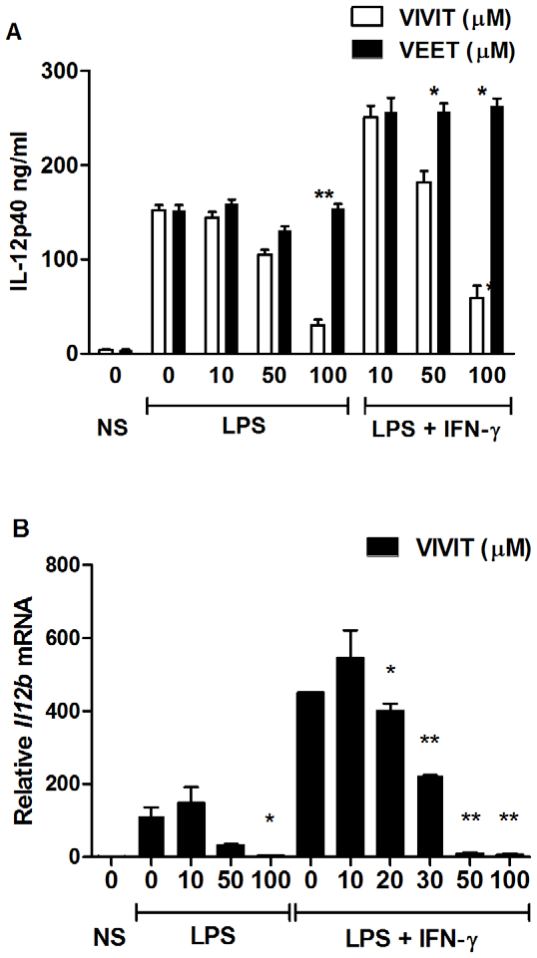
11R-VIVIT inhibits IL-12 p40 protein and mRNA expression. (**A**) Murine bone marrow-derived macrophages (BMDMs) were either untreated or pretreated with the indicated concentrations of 11R-VIVIT or the control peptide 11R-VEET for 1 h followed by LPS (100 ng/ml) alone or together with IFN-γ (10 ng/ml) for 24 h. IL-12 p40 protein secretion was assayed from supernatants by ELISA. (**B**) BMDMs were either untreated or pretreated with the indicated concentrations of 11R-VIVIT for 1 h followed by 1 h treatment with IFN-γ (10 ng/ml) prior to LPS (100 ng/ml) treatment for 4 h. Cells were harvested and total RNA was assayed for *Il12b* mRNA levels by real-time RT-PCR. Results were normalized with the respect to the levels of *β-actin* mRNA, and represent the mean ± SE for duplicate assays from three independent experiments. * p<0.05, ** p<0.01.

Tacrolimus inhibits calcineurin activation and may have effects on other important signal transduction pathways such as NF-κB [18], [31]. To validate these findings and importantly, to demonstrate specificity for NFAT activation, we utilized the cell permeable peptide 11R-VIVIT. In LPS and LPS plus IFN-γ stimulated BMDMs, 11R-VIVIT dose dependently attenuated IL-12 p40 protein secretion (Figure 2A) and *Il12b* mRNA expression (Figure 2B). The inactive control peptide 11R-VEET did not inhibit IL-12 p40 production in LPS or LPS and IFN-γ activated BMDMs (Figure 2A).

### NFAT regulates the inflammatory cytokines IL-12 p70, IL-23 and TNF

**Figure 3:**
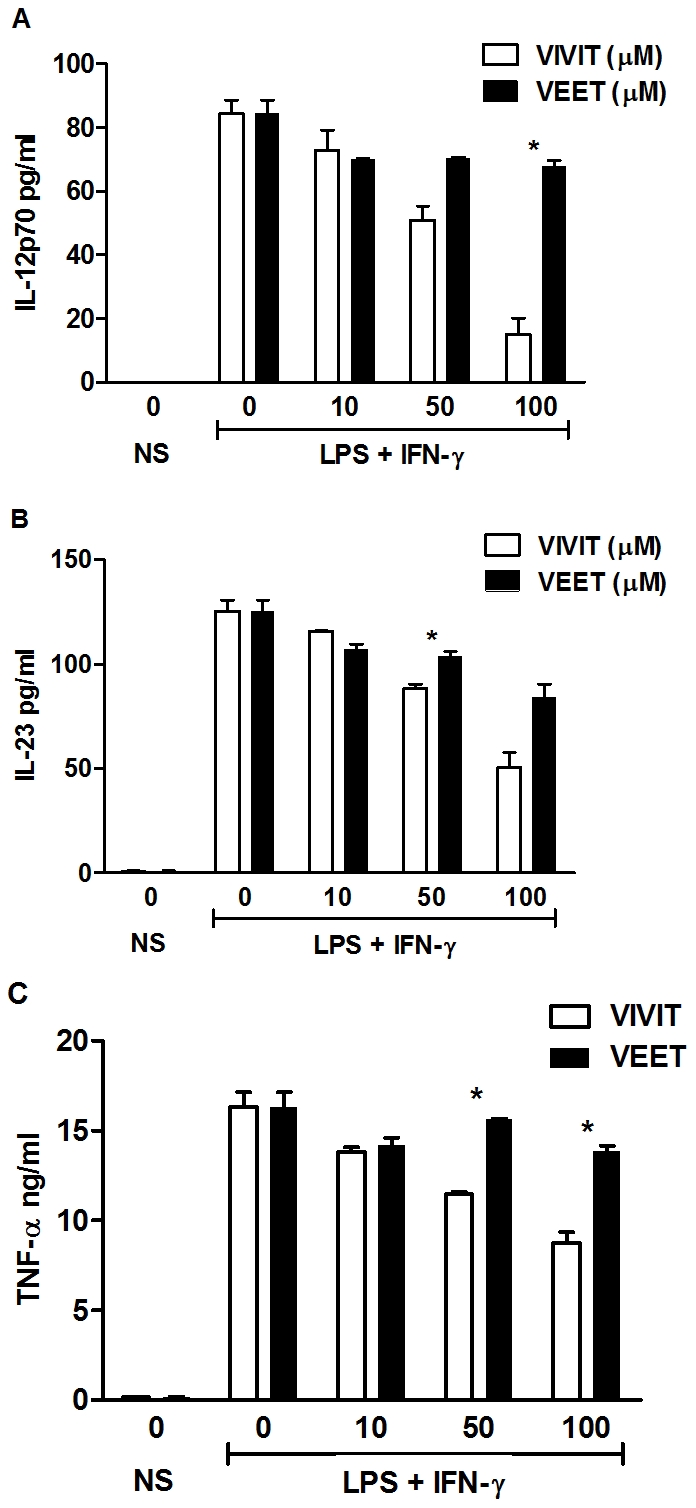
11R-VIVIT inhibits pro-inflammatory cytokine expression in murine macrophages. BMDMs from WT mice were either untreated or pretreated with the indicated concentrations of 11R-VIVIT or 11R-VEET for 1 h followed by LPS (100 ng/ml) and IFN-γ (10 ng/ml) for 24 h. IL-12 p70 (**A**), IL-23 (**B**) and TNF (**C**) protein secretion were assayed from supernatants by ELISA. Each result represents mean ± SE of duplicate assays from three independent experiments. * p<0.05.

IL-12 p40 heterodimerizes with the IL-12 p35 and IL-23 p19 subunits, respectively, to form the biologically active cytokines IL-12 p70 and IL-23. In BMDMs stimulated with LPS plus IFN-γ, 11R-VIVIT inhibited IL-12 p70 (Figure 3A) and IL-23 (Figure 3B) in a dose-dependent manner, while the control 11R-VEET peptide had no effect. Regulation of TNF by NFAT in T cells, B cells and myelomonocytes has been described in previous studies [32], [33], [34]. 11R-VIVIT suppressed the induction of TNF by LPS plus IFN-γ, while the control 11R-VEET did not (Figure 3C).

### 11R-VIVIT inhibits NFAT DNA binding to the murine *Il12*b promoter

**Figure 4:**
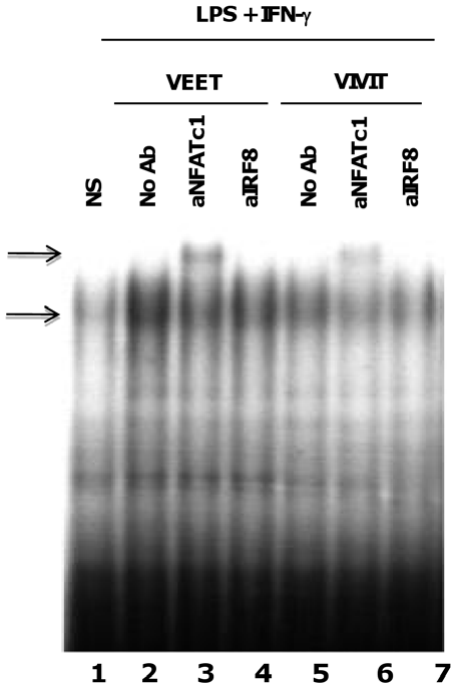
11R-VIVIT reduces DNA binding to the NFAT/IRF8 site in the murine IL-12 p40 promoter. BMDMs were either untreated (lane 1) or pretreated with the indicated concentrations of 11R-VEET (lanes 2–4) or 11R-VIVIT (lanes 5–7) for 1 h. Cells were then treated for 1 h with IFN-γ (10 ng/ml) prior to LPS (100 ng/ml) stimulation for 4 h. Cells were harvested and nuclear extracts were prepared for EMSA. ^32^P labeled oligonucleotide probe spanning the NFAT/IRF8 site of the IL-12 p40 promoter [10] was incubated with 10 mg of nuclear extracts on ice for 30 min prior to electrophoresis. For supershift assays (lanes 3,4 and 6,7), 10 mg nuclear extract were incubated with 3 mg of anti-NFAT c1 (lanes 3 and 6) or anti-IRF8 (lanes 4 and 7) antibodies for 30 min on ice prior to addition of the ^32^P labeled probe and 30 min incubation on ice followed by electrophoresis. The arrows represent DNA-protein complexes formed before and after incubation with the indicated antibodies. The above result is representative of five independent experiments.

We next analyzed DNA binding of NFAT to the NFAT/IRF8 site in the *Il12b* promoter by EMSA using nuclear extracts obtained from murine BMDMs. Activation of BMDMs with LPS and IFN-γ induced DNA-protein complex formation on an oligonucleotide probe containing the NFAT/IRF8 element, spanning the region from −88 to −54 (with respect to the transcription start site) of the murine *Il12b* promoter (Figure 4, lane 2). The presence of NFAT and IRF8 in this complex was confirmed by supershift using anti-NFAT and anti-IRF8 antibodies. Anti-NFAT antibody generated a supershift whereas anti-IRF8 antibody abrogated complex formation, as shown previously [10]. Treatment with 11R-VIVIT reduced DNA-protein complex formation in LPS and IFN-γ stimulated BMDMs (Figure 4, compare lanes 2 and 5). Supershifted bands showed reduced amounts of NFAT (Figure 4, compare lanes 3 and 6) and IRF8 (Figure 4, compare lanes 4 and 7). Furthermore, treatment of cells with the control 11R-VEET peptide did not affect DNA-protein complex formation (Figure 4, lanes 2–4). The above results demonstrate that the VIVIT peptide prevented nuclear translocation of NFAT leading to reduced DNA-protein complex formation in vitro. In addition, these results suggest that VIVIT inhibits IRF8 DNA binding as well, consistent with our previous model where NFAT binding recruits IRF8 to the Il12b promoter [10].

### NFAT is involved in inducible nitric oxide synthase (*Nos2*) expression

**Figure 5:**
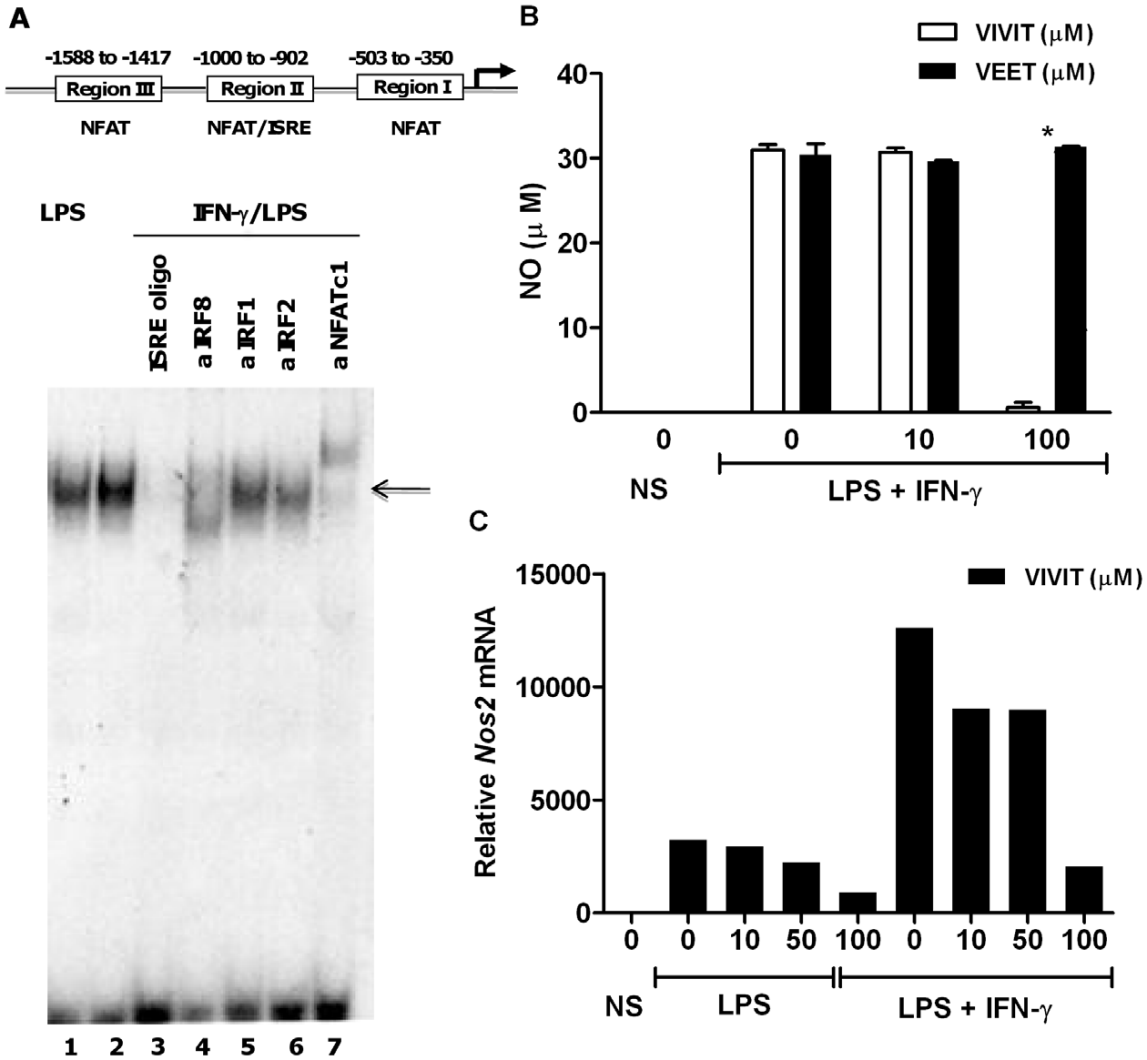
NFAT binds to *Nos2* promoter and its selective inhibition abrogates nitric oxide secretion in macrophages. (**A**) Nuclear extracts were prepared from BMDMs stimulated with LPS (lane 1) or LPS and IFN-γ (lanes 2–7). Extracts were either incubated with a labeled probe NFAT/ISRE (element from region II of the promoter scheme), a competitor ISRE oligonucleotide, or with the indicated antibodies. DNA-protein complexes (indicated by arrow) were separated by electrophoresis. EMSA revealed binding of both IRF8 and NFATc1 to the same *Nos2* promoter element. (**B**) BMDMs from WT mice were either untreated or pretreated with the indicated concentrations of 11R-VIVIT or the control peptide for 1 h followed by LPS (100 ng/ml) and IFN-γ (10 ng/ml) for 24 h. Nitric oxide secretion was assayed from supernatants by Greiss reaction. Experiments were performed in duplicate and repeated three times (mean ± SEM). (**C**) BMDMs were either untreated or pretreated with the indicated concentrations of 11R-VIVIT for 1 h followed by 1 h treatment with LPS alone or IFN-γ (10 ng/ml) prior to LPS (100 ng/ml) treatment for 4 h. Cells were harvested and total RNA was assayed for *Nos2* mRNA levels by real-time RT PCR. Data is representative of three independent experiments. * p<0.05.

We previously reported that IRF8 and IRF1 are essential for induction of the *Nos2* gene in macrophages [35]. Furthermore, *Nos2* regulation by calcineurin was also demonstrated in intestinal epithelial and macrophage cell lines [36], [37]. Based on these data, we postulated that the interaction between IRF8 and NFAT demonstrated on the *Il12b* promoter is likely to be relevant for expression of other genes. Through a database search, we identified a putative composite ISRE-NFAT binding site in the murine *Nos2* promoter [38] (Figure 5A). DNA binding of NFAT and IRF8 was next analyzed by EMSA using nuclear extract from BMDMs stimulated with LPS and IFN-γ. Supershift experiments demonstrate the presence of NFAT and IRF8 in the same protein complex bound to a probe spanning the ISRE binding element (Figure 5A, lanes 4 and 7). To examine the role of NFAT in Nos2 expression, we assayed nitric oxide production (measured as nitrite accumulation) by BMDMs treated with 11R-VIVIT. Inhibition of NFAT attenuated nitric oxide production (Figure 5B) and *Nos2* mRNA expression (Figure 5C) in a dose dependent manner. Thus, these results suggest that NFAT mediates the expression of *Nos2* and nitric oxide production in BMDMs.

### Inhibition of IL-12 p40 by VIVIT is independent of IL-10

**Figure 6:**
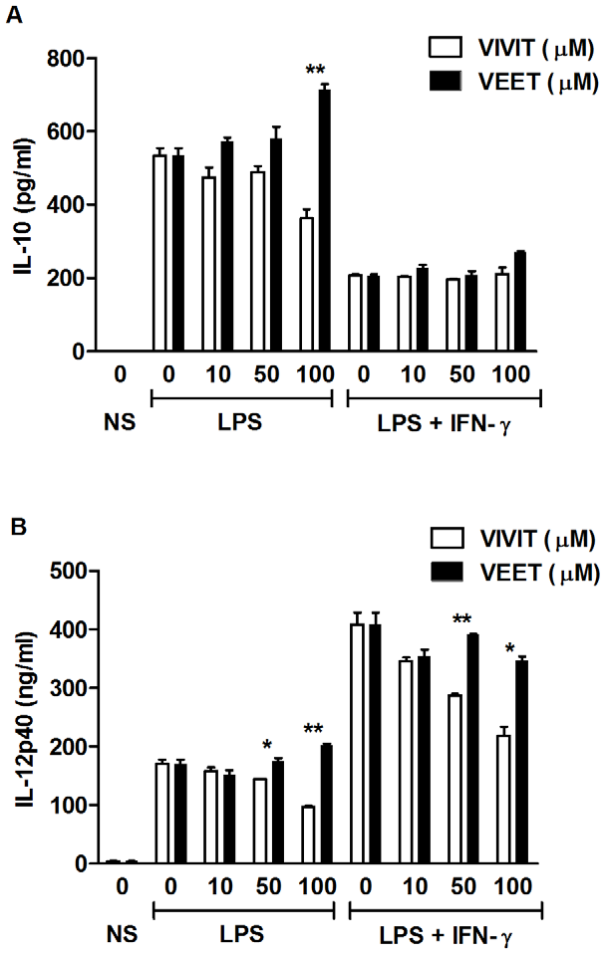
Inhibition of IL-12 p40 by 11R-VIVIT is independent of IL-10. (**A**) Murine BMDMs were either untreated or pretreated with the indicated concentrations of 11R-VIVIT (white bars) or 11R-VEET (black bars) for 1 h followed by LPS (100 ng/ml) alone or together with IFN-γ (10 ng/ml) for 24 h. IL-10 protein secretion was assayed from supernatants by ELISA. (**B**) BMDMs from *Il10* ^−/−^ mice were either untreated or pretreated with the indicated concentrations of 11R-VIVIT or 11R-VEET for 1 h followed by LPS (100 ng/ml) and LPS plus IFN-γ (10 ng/ml) for 24 h. IL-12 p40 protein secretion was assayed from supernatants by ELISA. Results represent the mean ± SD for duplicate assays from three independent experiments. * p<0.05, ** p<0.01.

IL-10 is an anti-inflammatory cytokine which inhibits macrophage activation and the pro-inflammatory response [39]. Importantly, IL-10 inhibits IL-12 p40 transcription [40]. Therefore, we determined whether inhibition of IL-12 p40 by VIVIT was mediated by induction of IL-10. IL-10 levels were evaluated in cell free supernatants of BMDMs treated with either 11R-VIVIT or control 11R-VEET (Figure 6A). Inhibition of NFAT did not significantly affect IL-10 in LPS or LPS plus IFN-γ-treated BMDMs. Furthermore, compared to the control peptide, 11R-VIVIT inhibits IL-12 p40 in BMDMs derived from *Il10^−/−^* mice in a dose dependent manner (Figure 6B), substantiating that VIVIT inhibits IL-12 p40 expression through IL-10 independent pathways.

### Inhibition of IL-12 p40 is not induced through disruption of TLR4 signaling

To verify that 11R-VIVIT does not inhibit LPS induced IL-12 p40 production through disruption of the LPS-TLR4 signaling pathway, we studied the effects of 11R-VIVIT^EMD^ on LPS stimulated BMDMs isolated from *Il10^−/−^*;NF-κB^EGFP^ mice. The percentage of GFP positive cells was similar for LPS activated BMDMs (8.5±0.97%) exposed to VIVIT (9.2±2.2%) and VEET (9±0.96%), all significantly higher (P<0.05) compared to cells that were exposed to PBS alone (1.79±0.1%), suggesting that 11R-VIVIT does not inhibit LPS induced NF-κB activation (Figure S1, representative images).

### Administration of 11R-VIVIT ameliorates experimental colitis

We postulated that blocking the calcineurin/NFAT pathway with 11R-VIVIT could ameliorate IL-12/23 mediated experimental colitis. We examined the effects of 11R-VIVIT administration on the course of colitis in piroxicam treated *Il10^−/−^* mice, a commonly used murine IBD model characterized by increased colonic *Il12b* expression [29]. The cyclooxygenase 1 and 2 inhibitor, piroxicam (200 ppm), was added to the diet of 5 weeks old mice for 14 days as a validated approach to accelerate the development and increase the penetrance of colitis in *Il10^−/−^* mice [27].

**Figure 7:**
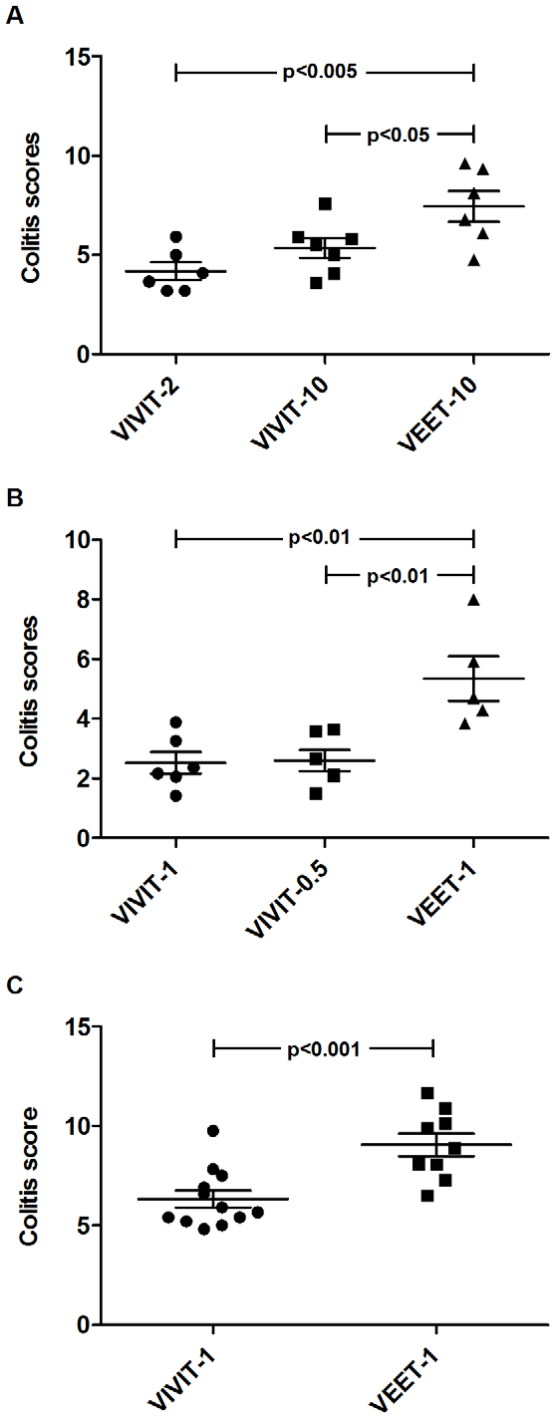
Therapeutic effect of VIVIT on piroxicam induced colitis in *Il10* ^−/−^ mice. Piroxicam fed IL-10 deficient mice were injected with the indicated doses of either VIVIT or VEET peptides (6–7 mice per group) every other day for two weeks. Colons were then harvested and colitis scores were determined as described in materials and methods. (**A–B**) An inverse dose response of VIVIT therapy was noted, with a higher mortality rate at 10 mg/kg (**A**) and amelioration of colitis detected at as low as 0.5 mg/kg following one week of piroxicam diet (**B**). (**C**) 1 mg/kg of either peptide was then administered to a group of 21 piroxicam fed *Il10* ^−/−^ mice. The scores for histological damage during piroxicam-induced colitis were significantly higher in VEET treated mice compared to those receiving VIVIT peptide in all reported experiments. Data shown are mean ± SEM.

High doses of 11R-VIVIT (2 and 10 mg/kg) decreased colitis scores significantly (Figure 7A) compared to an inactive cell permeable control peptide (11R-VEET). However, high doses of VIVIT may also inhibit T regulatory cell populations [41] and consequently increase inflammation. Consequently we repeated the experiment (Figure 7B) and demonstrate amelioration of colitis at doses as low as 0.5 mg/kg. To confirm the therapeutic effects of VIVIT at low dosage, a large cohort of mice (n = 21) were treated with 1 mg/kg of 11R-VIVIT or 11R-VEET every other day for two weeks following 14 days of piroxicam administration. Administration of 11R-VIVIT demonstrated therapeutic efficacy as measured by increases in body weight and gross colonic appearance. Colons from VIVIT treated mice showed formed stool pellets and decreased wall thickening (Figure S2). Consistent with macroscopic observations, histological examination showed reduced numbers of infiltrating cells within the submucosa and lamina propria in the VIVIT treated mice compared to VEET treated ones (Figure S2B). Epithelial damage and hyperplasia were significantly reduced in VIVIT treated mice (Figure S2B). Furthermore, a significant decrease in histological scores in mice receiving 11R-VIVIT compared to those receiving 11R-VEET was observed in 3 independent experiments (Figure 7C).

### Treatment with 11R-VIVIT reduces colonic secretion of pro-inflammatory cytokines

**Figure 8:**
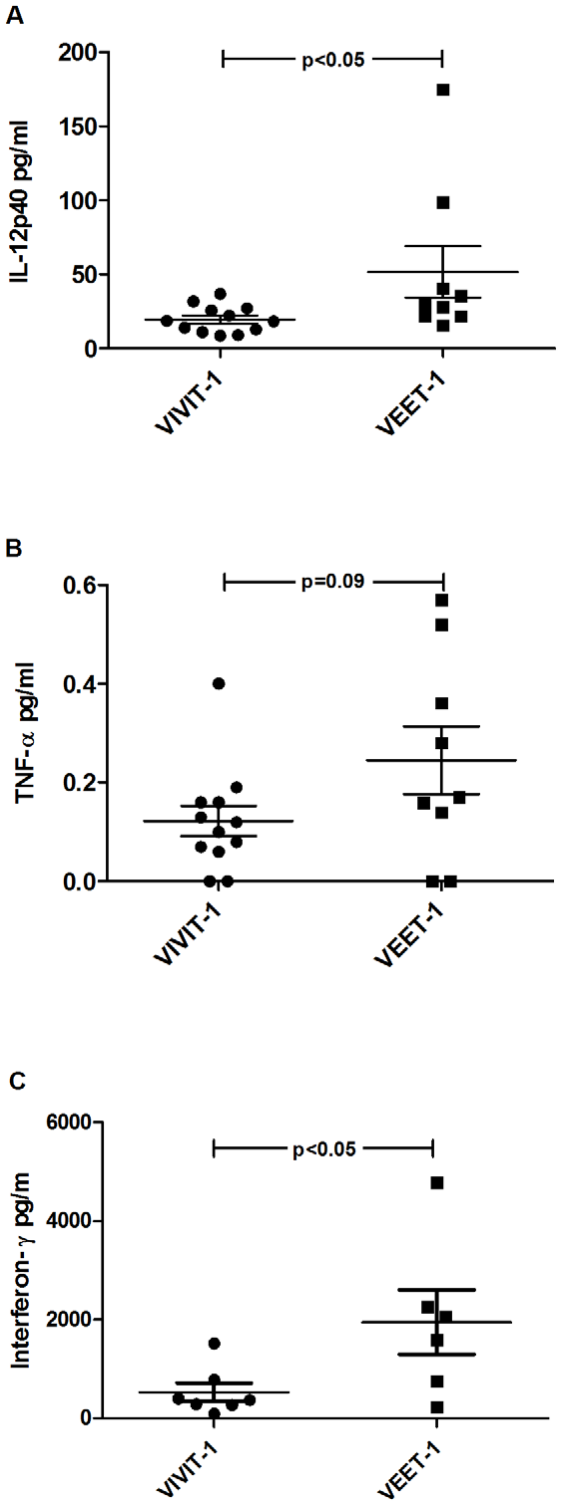
VIVIT treatment reduces spontaneous secretion of colonic inflammatory cytokines. Explants from colons of VIVIT (n = 12 for **A**, **B**; n = 7 for **C**) and VEET (n = 9 for **A**, **B**; n = 6 for **C**) treated mice were incubated in RPMI medium for 24 h, released cytokines were then determined by ELISA. We noted a marked difference in the gut spontaneous secretion of TNF-α (**B**) in gut supernatants from VIVIT treated mice, with a statistically significant change in IL-12 p40 (**A**) and in interferon-γ (**C**) production compared to VEET treated mice. Values are normalized to weight of intestinal explants, and data are presented as mean ± SEM.

We next investigated whether the expression of colonic pro-inflammatory cytokines correlated with histologic improvement in 11R-VIVIT treated *Il10^−/−^* mice. 11R-VIVIT treatment in vivo significantly down-regulated the production of IL-12 p40 (19.6±2.65 pg/ml versus 51.87±17.43 pg/ml in controls; p = 0.048) and IFN-γ levels (533±182 pg/ml vs 1956±650 pg/ml; p = 0.046); while TNF levels were marginally decreased (Figure 8) in colonic explant cultures. As IFN-γ is the signature Th1 cytokine downstream of IL-12 signaling, decreased colonic IFN-γ may be a consequence of lower IL-12 levels in vivo, but VIVIT may also have direct effects on inflammatory T cells, as previously demonstrated [23]. Indeed, 11R-VIVIT directly inhibited release of IFN-γ from anti-CD3/CD28 activated T cells in culture (Figure S3).

## Discussion

In this study, we demonstrate that intracellular delivery of 11R-VIVIT inhibited expression and secretion of inflammatory cytokines in macrophages and ameliorated active colitis in piroxicam treated *Il10^−/−^* mice. Thus, modulation of NFAT activity by this cell permeable peptide could be an effective therapeutic strategy in inflammatory bowel disease.

The NFAT family is a complex family of transcription factors with five proteins, each member having different alternatively spliced isoforms. NFAT is important for the development and function of numerous major organ systems including the nervous system, heart, blood vessels, kidney, bone and muscle [13]. NFAT transcription factors play an important role in regulating immune/inflammatory signaling, with the best described effects on T cell activation and phenotype. NFAT also regulates gene expression in other immune cells such as B cells [42], mast cells [43], eosinophils [44], basophils [45] and NK cells [46]. In dendritic cells, LPS stimulation resulted in Ca^2+^ influx and NFAT activation that regulated their life cycle [15]. NFAT proteins are involved in the differentiation of monocyte/macrophage lineage cells into osteoclasts [47]. However, knowledge of NFAT function specifically in macrophages is limited. Macrophages express NFAT family members [10], [47] and in our previous study, we demonstrated the requirement for NFATc1 and c2 in Il12b gene expression in a murine macrophage cell line [10]. Ca^2+^/calcineurin signaling has been implicated in macrophage biology, with conflicting results reported. Conboy and colleagues showed that Ca^2+^/calcineurin signaling inhibited macrophage cytokine production [48]. However, a later study reported that calcineurin signaling induced *Nos2* expression and NO secretion in murine macrophages [49]. Moreover, the African swine fever virus protein, A238L has been shown to inhibit inflammatory gene expression in infected macrophages by binding to calcineurin and inhibiting NFAT-dependent gene expression [50]. Furthermore, in a study by Jeffrey et al. [51], macrophages deficient for the dual specificity phosphatase- PAC-1, displayed decreased activation of NFAT and diminished expression of pro-inflammatory mediators.

Here, we show that NFAT is important for pro-inflammatory gene expression by murine macrophages using the selective inhibitory peptide VIVIT. NFAT regulates the expression of the *Il12b* gene, thereby controlling the levels of the bioactive heterodimers of IL-12 p70 and IL-23. We also show that NFAT regulates TNF and *Nos2* expression in BMDMs. Although an NFAT binding site has been reported within the *Tnf* gene in T cells [52], [53], the importance of this element is far less established in macrophages and thus, it is unclear whether VIVIT-dependent reduction in TNF secretion is mediated through direct inhibition of NFAT recruitment to the *Tnf* gene or through indirect effects (eg, post-transcriptional regulation) in cells and/or a general reduction in inflammation in vivo. Importantly, we show that VIVIT-dependent inhibition of LPS-induced inflammatory mediator secretion is not through inhibition of the canonical TLR4 signaling pathway, as VIVIT does not abrogate NF-κB activation in BMDMs. Thus, NFAT, functioning as a complex with IRF family members or other interacting partners has a general yet underappreciated role in programming macrophage gene expression. In support of our findings, it has been reported that VIVIT effectively inhibited LPS induced secretion of TNF and MCP-1 in murine microglia cultures [54].

Moreover, VIVIT administration in vivo ameliorates colitis in *Il10^−/−^* mice and histologic improvement correlated with decreased colonic IL-12 p40 secretion. Although, VIVIT at 10 mg/kg was effective in amelioration of colitis, we show that doses as low as 0.5 mg/kg were effective. These data suggest that VIVIT treatment contributed to the amelioration of piroxicam induced colitis in *Il10* ^−/−^ mice, in part by decreasing pro-inflammatory cytokine release by macrophages. However, NFAT has an important role in regulation of T cells, and since *Il10^−/−^* colitis model is driven by the innate and adaptive immune system, we cannot exclude inhibitory effects of VIVIT on T cells as an additional mechanism for amelioration of colitis. Indeed, NFAT proteins are involved in the induction and regulation of Th1, Th2 and Th17 responses. In fact, NFATc1 and c2 deficient mice display impaired Th1 and Th2 response [55], [56], and NFATc2 deficiency has been reported to suppress colitis induced by oxazolone administration [57]. Furthermore, sustained NFATc1 signaling has been shown to promote a Th1-like pattern of gene expression in murine CD4^+^ T cells [58]. Accordingly, we found that colonic IFN-γ levels were reduced in *Il10^-/-^* mice exposed to 11R-VIVIT in vivo. As IFN-γ is the signature IL-12 induced Th1 cytokine, this phenomenon may be a consequence of decreased IL-12 in vivo, but VIVIT may also have direct effects on inflammatory T cells, as previously demonstrated [23] and confirmed in this study (Figure S3). Moreover, NFAT was shown to be important for the induction of Th17 signature cytokines , IL-17, IL-21 and IL-22 [59]. NFAT2 was also reported to play vital role in FoxP3 transcription and hence T regulatory cell expansion [41], [60], [61]. Interestingly, exposing mice to higher doses of VIVIT in vivo may not be as effective in ameliorating colitis as lower concentrations, as is suggested by our experimental results (Figure 7), due to inhibition of T regulatory cell activation or expansion.

Additional NFAT-driven mechanisms that may contribute to the decrease in chronic intestinal inflammation may include effects on dendritic cells. Zanoni et al [15] have shown that exposure of dendritic cells to LPS results in apoptosis which is dependent on NFAT. Blocking of this pathway may result in the maintenance of dendritic cells with regulatory properties. Intestinal dendritic cells have a prominent role in tolerance induction towards bacterial antigens. In addition Jennings et al [62] have shown that calcineurin inhibition leads to decreased responsiveness (tolerance) to LPS in macrophages and dendritic cells. This finding may suggest an alternative mechanism of immune response down-regulation towards enteric bacteria and bacterial products.

There are now numerous examples of PTD-containing peptides having significant effects in preclinical models of inflammatory disease [63], [64]. Although the mechanisms through which PTDs deliver their cargo intracellularly are not completely delineated, cells specialized to sample the environment such as macrophages and dendritic cells may be preferential targets for PTDs (as opposed to small molecules).

The immunosuppressive agents CsA and tacrolimus inhibit the phosphatase activity of calcineurin, thus preventing nuclear localization of NFAT [65]. However, the use of CsA and tacrolimus in chronic inflammatory disorders is limited by toxicities, including chronic nephro- and neurotoxicity among other significant side effects [66], [67]. These agents also inhibit other pathways downstream of calcineurin which could lead to undesired effects [66], [67]. Unlike tacrolimus and CsA, VIVIT will not affect calcineurin activity, which speculatively poses safety advantages in human disease. Other non-NFAT related mechanisms have been described for calcineurin inhibitors. Relevant to this study, in a human promonocytic cell-line, calcineurin inhibitors regulated LPS-induced IL-12 p40 expression through a mechanism that involved NF-κB [18].

In conclusion, these experiments demonstrate that NFAT plays an important role in inflammatory gene expression in macrophages, and a specific short peptide inhibitor of NFAT dephosphorylation, VIVIT, attenuates inflammation in a murine model of experimental colitis. Cell permeable 11R-VIVIT peptide could represent a novel therapeutic approach in chronic inflammatory diseases where both innate and adaptive immune responses play a role in pathogenesis.

## Supporting Information

Figure S1 **Inhibition of IL-12 p40 is not induced through disruption of TLR4 signaling.** BMDMs isolated from *Il10^−/−^*;NF-κB^EGFP^ mice were exposed to 11R-VIVIT^EMD^ or 11R-VEET for 1 hour prior to exposure to ultrapure LPS (100 ng/ml); controls were exposed to LPS or PBS. Representative images of high power field magnification ×40 of GFP positive BMDMs overlayed on total BMDMs. (**A**) PBS stimulated BMDMs. (**B**) LPS stimulated BMDMs. (**C**) VEET and LPS stimulated BMDMs. (**D**) VIVIT and LPS stimulated BMDMs. The experiment was repeated on BMDM preparations from 3 different mice with similar results.
(TIF)

Figure S2 **Inflammation is attenuated following treatment with 11R-VIVIT.** (**A**) Representative photograph of colons from VIVIT and VEET treated *Il10* ^−/−^ as indicated. Colon from VIVIT treated mice show a clear decrease in tissue thickening with well-formed stool pellets compared to those from VEET treated mice. (**B**) Colons of piroxicam treated *Il10* ^−/−^ mice were evaluated for pathology following two weeks of either VIVIT or VEET injections. H&E stained colon tissue from VIVIT treated mice show mild signs of inflammation and epithelial damage compared to that from VEET treated mice. VIVIT treatment protected piroxicam treated *Il10* ^−/−^ mice from epithelial hyperplasia, crypt destruction, and inflammatory invasion in both mucosa and sub-mucosa. Representative histological sections of colons from treated and control mice are shown (magnification ×10).
(TIF)

Figure S3 **11R-VIVIT inhibits activated T cell cytokine secretion.** Levels of IFN-γ were assessed as a measure of an activated Th1 response in cells. Splenic CD4+ T cells from WT mice were stimulated with plate bound anti-CD3 (5 µg/ml) and anti-CD28 (2 µg/ml) (eBioscience) and exposed to various concentrations of 11R-VIVIT^EMD^ or VEET. Cells were harvested after 4 hours for mRNA and supernatants were collected after 48 hours for ELISA. IFN-γ mRNA (**A**) and protein (**B**) were dose dependently reduced by11R-VIVIT^EMD^.
(TIF)
