# Supplementary figures and images for: A Cell Permeable Peptide Inhibitor of NFAT Inhibits Macrophage Cytokine Expression and Ameliorates Experimental Colitis
Source: PLoS One. 2012 Mar 27;7(3):e34172. doi: 10.1371/journal.pone.0034172 (PMC3313977; doi:10.1371/journal.pone.0034172)

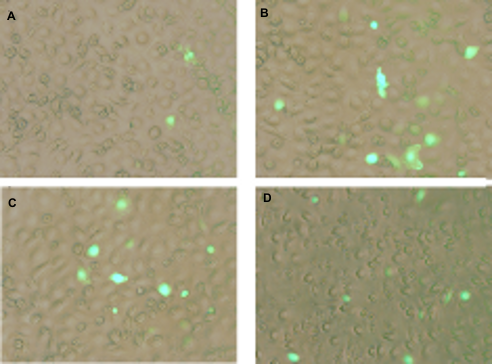

Supplement: Figure S1 — Inhibition of IL-12 p40 is not induced through disruption of TLR4 signaling. BMDMs isolated from Il10−/−;NF-κBEGFP mice were exposed to 11R-VIVITEMD or 11R-VEET for 1 hour prior to exposure to ultrapure LPS (100 ng/ml); controls were exposed to LPS or PBS. Representative images of high power field magnification ×40 of GFP positive BMDMs overlayed on total BMDMs. (A) PBS stimulated BMDMs. (B) LPS stimulated BMDMs. (C) VEET and LPS stimulated BMDMs. (D) VIVIT and LPS stimulated BMDMs. The experiment was repeated on BMDM preparations from 3 different mice with similar results. (TIF) [file pone.0034172.s001.tif]

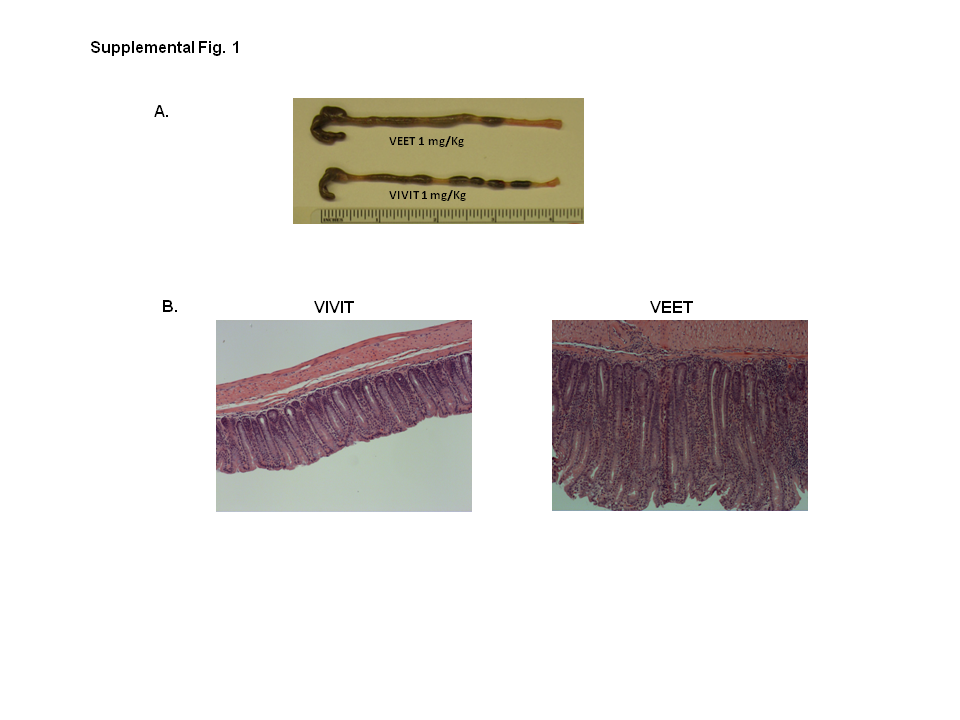

Supplement: Figure S2 — Inflammation is attenuated following treatment with 11R-VIVIT. (A) Representative photograph of colons from VIVIT and VEET treated Il10 −/− as indicated. Colon from VIVIT treated mice show a clear decrease in tissue thickening with well-formed stool pellets compared to those from VEET treated mice. (B) Colons of piroxicam treated Il10 −/− mice were evaluated for pathology following two weeks of either VIVIT or VEET injections. H&E stained colon tissue from VIVIT treated mice show mild signs of inflammation and epithelial damage compared to that from VEET treated mice. VIVIT treatment protected piroxicam treated Il10 −/− mice from epithelial hyperplasia, crypt destruction, and inflammatory invasion in both mucosa and sub-mucosa. Representative histological sections of colons from treated and control mice are shown (magnification ×10). (TIF) [file pone.0034172.s002.tif]

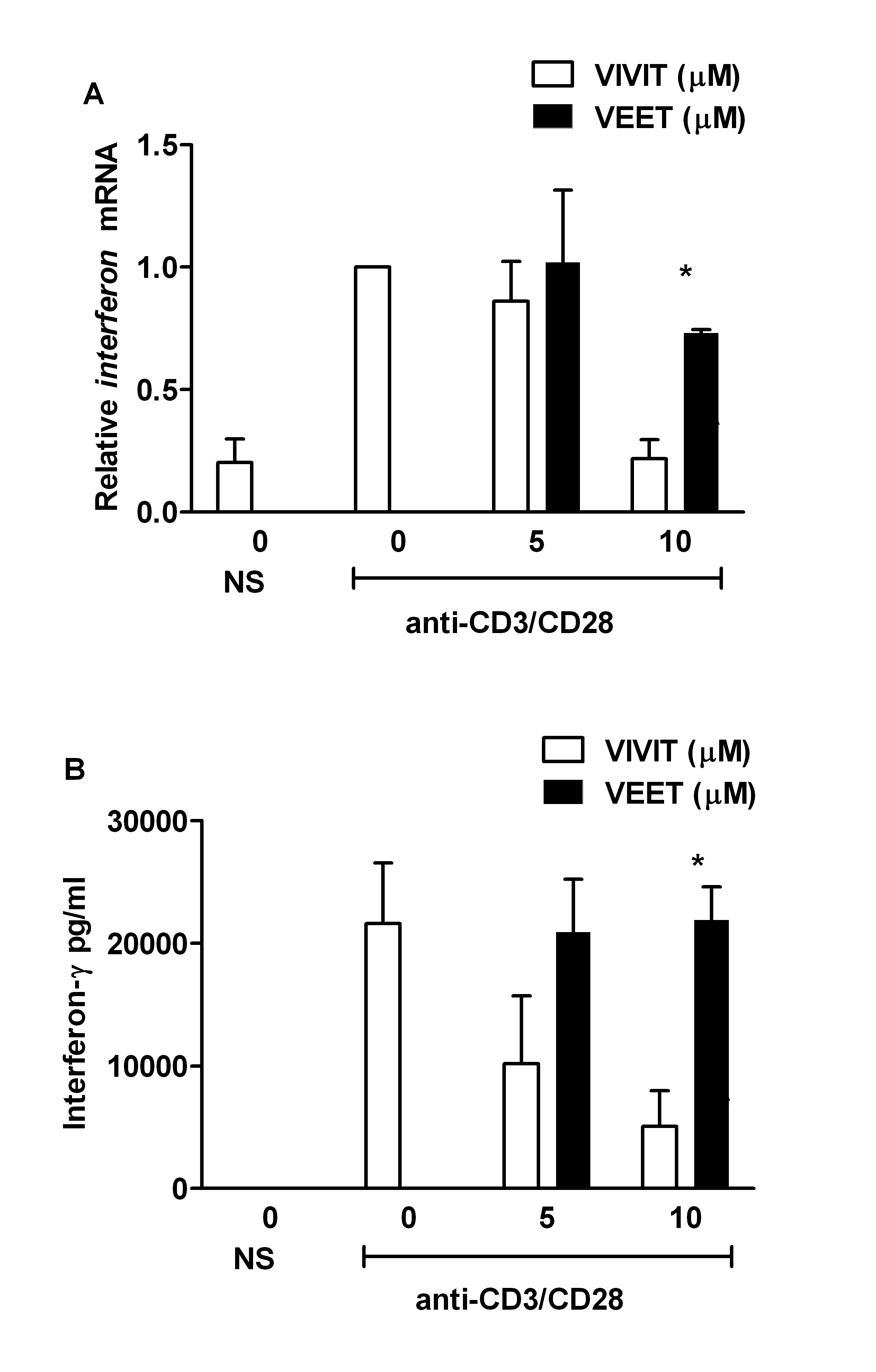

Supplement: Figure S3 — 11R-VIVIT inhibits activated T cell cytokine secretion. Levels of IFN-γ were assessed as a measure of an activated Th1 response in cells. Splenic CD4+ T cells from WT mice were stimulated with plate bound anti-CD3 (5 µg/ml) and anti-CD28 (2 µg/ml) (eBioscience) and exposed to various concentrations of 11R-VIVITEMD or VEET. Cells were harvested after 4 hours for mRNA and supernatants were collected after 48 hours for ELISA. IFN-γ mRNA (A) and protein (B) were dose dependently reduced by11R-VIVITEMD. (TIF) [file pone.0034172.s003.tif]
